# Sirtinol, a Sir2 protein inhibitor, affects stem cell maintenance and root development in *Arabidopsis thaliana* by modulating auxin-cytokinin signaling components

**DOI:** 10.1038/srep42450

**Published:** 2017-02-14

**Authors:** Sharmila Singh, Alka Singh, Sandeep Yadav, Vibhav Gautam, Archita Singh, Ananda K. Sarkar

**Affiliations:** 1 National Institute of Plant Genome Research, Aruna Asaf Ali Marg, New Delhi 110067 India

## Abstract

In *Arabidopsis thaliana,* besides several key transcription factors and chromatin modifiers, phytohormones auxin and cytokinin play pivotal role in shoot and root meristem maintenance, and lateral root (LR) development. Sirtinol, a chemical inhibitor of Sir2 proteins, is known to promote some auxin induced phenotypes in *Arabidopsis.* However, its effect on plant stem cell maintenance or organ formation remained unaddressed. Here we show that sirtinol affects meristem maintenance by altering the expression of key stem cell regulators, cell division and differentiation by modulating both auxin and cytokinin signaling in *Arabidopsis thaliana*. The expression of shoot stem cell niche related genes *WUSCHEL (WUS)* and *CLAVATA3 (CLV3)* was upregulated, whereas *SHOOT MERISTEMLESS (STM)* was downregulated in sirtinol treated seedlings. The expression level and domain of key root stem cell regulators *PLETHORA (PLTs)* and *WUS-Related Homeobox 5 (WOX5)* were altered in sirtinol treated roots. Sirtinol affects LR development by disturbing proper auxin transport and maxima formation, similar to 2,4-dichlorophenoxyacetic acid (2,4-D). Sirtinol also affects LR formation by altering cytokinin biosynthesis and signaling genes in roots. Therefore, sirtinol affects shoot and root growth, meristem maintenance and LR development by altering the expression of cytokinin-auxin signaling components, and regulators of stem cells, meristems, and LRs.

Unlike animals, plants continuously produce new organs throughout their lifetime through the meristematic activity maintained by stem cells that reside in shoot and root apical meristems (SAM and RAM). In *Arabidopsis*, the growth of both primary root and lateral roots (LRs) is maintained by the meristematic activity of RAM and LR meristem. SAM and RAM are maintained through the continuous supply of cell pool by the activity of stem cells that reside in the ‘stem cell niches’ of their respective meristems. In the stem cell niches, a few mitotically less active cells called as organizing center (OC) in SAM and quiescent center (QC) in RAM maintain the neighboring stem cell population through complex mutual signaling^1^. In SAM, *WUSCHEL (WUS*) expression in the OC induces the expression of *CLAVATA3 (CLV3)* in stem cells above, which in turn limits *WUS* expression and maintain stem cells or meristematic activity^2^,^3^,^4^. Other than WUS/CLV pathway, Class1 *KNOTTED LIKE HOMEOBOX (KNOX)* genes, which include *SHOOTMERISTEMLESS (STM), BREVIPEDICELLUS/KNAT1 (BP/KNAT1), KNAT2* and *KNAT6* are also involved in SAM maintenance^5^. STM represses the differentiation of SAM by inhibiting the expression of MYB related gene *ASYMMETRIC LEAVES 1 (AS1)* in stem cells, which in turn inhibits the expression of *KNAT1* and *KNAT2* in lateral organ primordia^6^.

A pathway partially similar to WUS/CLV acts in RAM maintenance, where QC plays important role in stem cell maintenance^7^. A homolog of WUS, *WUS-RELATED HOMEOBOX 5 (WOX5*), is expressed in QC and is required for the maintenance of columella stem cells (CSCs) and proximal stem cells, where it works along with *SCARECROW (SCR), SHORT-ROOT (SHR*) and *PLETHORA (PLT*) genes^7^,^8^,^9^. SHR, SCR and PLT proteins are required for QC identity and meristem maintenance^10^,^11^,^12^.

Besides these transcription factors, phytohormones also play important role in meristem maintenance. In the RAM, auxin maxima are formed in the QC and some columella cells and stem cells, where auxin efflux carriers PIN FORMED (PIN) proteins play important role^13^,^14^. In the root stem cell niche, auxin function is mediated by the action of PLT proteins, which form a gradient from stem cell niche to elongation or differentiation zone^15^,^16^. On the other hand, cytokinin interacts with auxin in an antagonistic manner to regulate root development^17^,^18^. Auxin promotes cell division, whereas cytokinin activates differentiation process^17^. This antagonism of auxin and cytokinin involves a regulatory circuit, where ARABIDOPSIS RESPONSE REGULATOR 1 (ARR1) and ARR12 activate the expression of *SHORT HYPOCOTYL2 (SHY2*), an AUX/IAA protein, which in turn represses the expression of *PINs*, and in a negative feedback loop, PINs inhibit the expression of *SHY2*^17^,^18^. This balance of auxin and cytokinin ratio defines the RAM size, cell division and differentiation, and thereby regulate root growth.

The balance of auxin and cytokinin signaling is required not only to control RAM size but also LR development. In *Arabidopsis,* LR initiation is governed by the perception of oscillating auxin maxima by xylem pole pericycle (XPP) cells, also known as LR founder cells (LRFCs)^19^,^20^,^21^. Multiple AUX/IAA-ARF modules are also known to regulate LR initiation^22^. SOLITARY-ROOT (SLR)/IAA14-AUXIN RESPONSE FACTOR 7 (ARF7) - ARF19 module is involved in the regulation of nuclear migration and asymmetric division of founder cells during LR initiation^22^,^23^. It has been reported that exogenous application of indole-3- acetic acid (IAA), 1-naphthaleneacetic acid (NAA) and 2,4-D increased LR formation^24^. Developing LRPs accumulate auxin via polar auxin transport and inhibition of this transport by N-1- naphthylpthalamic acid (NPA) blocks LR formation^25^. In contrast to auxin, cytokinin negatively regulates LR formation^19^,^26^,^27^,^28^. Exogenous cytokinin treatment leads to inhibition of LR development by arresting cell division in the pericycle layer and altering *PINs* expression^19^,^29^. It has been shown that cytokinin deficient *CKX* transgenic plants are defective in LR spacing^30^. Cytokinin synthesized in LRFCs and neighboring pericycle cells (PCs) is involved in the maintenance of proper LR positioning, as evident by LR positioning defects observed in the higher order mutants of cytokinin biosynthesis genes^19^,^26^.

Using classical genetics approach, several auxin and cytokinin signaling genes involved in various developmental processes have been identified and studied for their functions. Apart from classical genetics, the chemical genetics approach uses cell permeable small molecules to disturb a gene function, similar to mutagenesis but in a rapid, reversible and conditional manner, and it has emerged as a powerful tool to study gene functions and characterize biological pathways^31^,^32^.

Sirtinol was identified as an inhibitor of silent information regulator (Sir2) family of proteins in a high throughput phenotypic screening of cells using ~1600 small molecules^33^. In the same study, it was found that sirtinol affects body axis formation and vascularization in *Arabidopsis,* a phenotype similar to *MONOPTEROS/AUXIN RESPONSE FACTOR 5 (MP/ARF5*) mutant^33^. Later on, sirtinol was reported to alter the expression pattern of auxin responsive reporter *DR5:GUS* and activate auxin signaling genes^34^. Sirtinol treatment caused rapid degradation of AXR3-NT-β-glucuronidase (GUS) fusion protein, suggesting that it activates auxin signaling by degrading negative regulators^34^. Sirtinol treatment causes several auxin-related developmental phenotypes such as adventitious root growth and inhibition of primary root elongation^34^. In different genetic screens, several auxin resistant mutants such as *axr1, axr2, axr3*, etc. were found to be sirtinol resistant, which further suggest that it affects auxin signaling pathway^34^,^35^.

Since sirtinol treated seedlings showed defective root and shoot development, we hypothesized that sirtinol might do so by affecting the stem cell or meristem maintenance. We addressed this by analyzing the meristem phenotype, and expression of different molecular regulators of stem cell niches or meristems. We found that sirtinol did affect the expression of molecular regulators of SAM and RAM, and LR development. We observed that besides activating auxin signaling, sirtinol also affected cytokinin biosynthesis and signaling in roots. Interestingly, our observation also suggests that sirtinol induced defective LR development is partially similar to 2,4-D treatment.

## Results

### Sirtinol affects both shoot and root growth, and gravitropism in *Arabidopsis*

**Figure 1:**
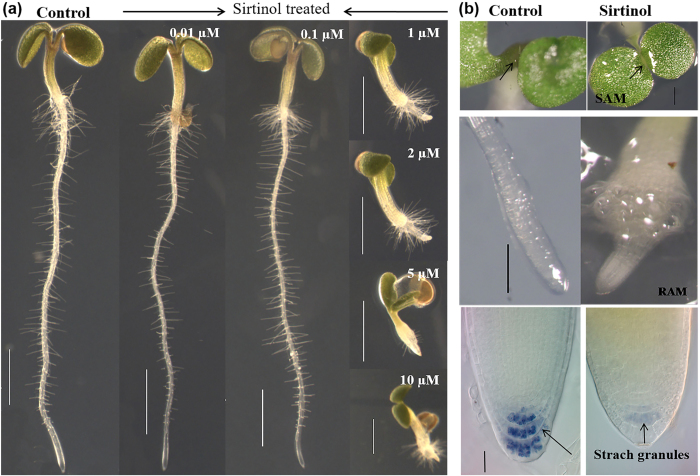
Sirtinol affects shoot and root development in a dose-dependent manner. (**a**) Sirtinol hinders plant growth in a dose dependent manner. Wild type seedlings were grown vertically on half MS media containing 0.01 μM, 0.1 μM, 1 μM, 2 μM, 5 μM, and 10 μM sirtinol. Phenotype was observed at 2 dag. Scale bar: 1 mm. (**b**) Sirtinol leads to defective SAM and RAM. Seedlings (at 2 dag) were visualized under stereomicroscope to study the effect of sirtinol (10 μM). Scale bar: 200 μm. Black arrows indicate accumulation of starch granules (Scale bar: 10 μm).

Previous reports showed that sirtinol treatment at concentration of 5 μM to 25 μM affected seedling growth in *Arabidopsis* in a manner partially similar to auxin^34^,^35^,^36^. Since phytohormones and inhibitors or activators are often known to work in a dose dependent manner, showing a range of phenotypic effect, we first examined the dose dependent effect of sirtinol on seedling development by growing them on ½ Murashige and Skoog (MS) media supplemented with 0–10 μM of sirtinol till two days after germination (2 dag). We observed that 0–0.1 μM sirtinol did not affect growth, however, as the concentration was increased to 1 μM and above (up to 10 μM), seedlings showed severe defects in both shoot and root growth (Fig. 1a). Sirtinol treated seedlings failed to develop proper shoots and leaf primordia, and roots were swollen and retarded (Fig. 1b).

We observed additional phenotype, such as loss of gravitropism, in sirtinol treated seedlings (Supplemental Fig. S1a). Based on their response to gravity, we categorized sirtinol treated seedlings, and observed that in a vertically grown plate, only ~25% seedlings showed positive gravitropism, roots of ~28% seedlings were facing upwards, 30% were growing horizontally, and roots of ~10% seedlings were tilted and approximately 5% had slightly less retarded roots (Supplemental Table S1). Less retarded root growth of a few seedlings could be caused by detachment of agravitropic root from sirtinol medium. We observed that accumulation of gravity sensing starch granules was reduced in columella of sirtinol treated roots (Supplemental Fig. S1b). These results suggest that sirtinol also affects the gravitropic response of the plants, besides effect on root growth. Together our results suggest that sirtinol affects growth and development of *Arabidopsis* seedlings.

### Sirtinol affects meristematic activities of both shoot and root

**Figure 2:**
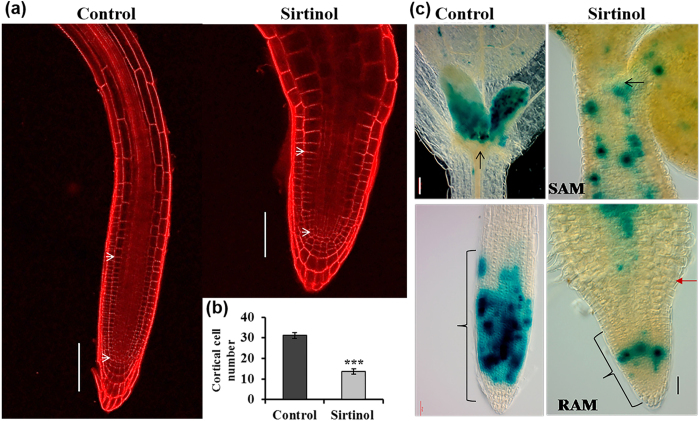
Effect of sirtinol treatment on root meristem size and cell division. (**a**) Sirtinol treatment reduced the root meristem size. To analyze root meristem size, number of cortical cells was quantified by counting from QC to first elongating cell (marked by white arrow heads) in control and sirtinol treated seedling at 2 dag. Scale bar: 100 μm. (**b**) Number of cortical cells is reduced in roots of sirtinol treated seedlings. Error bars indicate ± standard deviation (SD) (n = 20). One-way ANOVA was performed for statistical analysis. Asterisks indicate significant statistical differences, ***P < 0.001, **P < 0.01, *P < 0.05. Experiment was repeated 3 times with reproducible results. (**c**) Sirtinol affects cell division in meristems. To analyze cell division, *CyclinB1;1:CDB-GUS* marker seedlings were grown on sirtinol containing medium and staining for GUS was performed at 2 dag. Scale bar: 50 μm. Black arrows indicate SAM, brackets indicate RAM and bold red arrow marks root-shoot junction.

Shoot and root growth and organ patterning require maintenance and activity of their respective meristems. Since sirtinol treated roots were significantly smaller than control, we examined the RAM size in sirtinol grown seedlings at 2 dag (Fig. 2a). RAM size was calculated by quantifying cortical cell number from QC to the first elongating cell of RAM. We observed that the treatment with sirtinol reduced the RAM size. Sirtinol treated roots had reduced number of cortical cells (~13) in comparison to untreated control (~31), in the meristem region (Fig. 2a,b). The reduced number of cortical cells in root meristem of sirtinol grown seedlings suggests defective cell division progression. We, therefore, examined the expression pattern of *CyclinB1;1:CDB-GUS* reporter, which marks the G2/M phase transition of cell cycle, in the root tip of plants grown with or without sirtinol (Fig. 2c). We observed that the division of meristematic cells was drastically reduced and the dividing cells were randomly distributed in roots of sirtinol grown seedlings, as compared to control (Fig. 2c). These results suggest that sirtinol affects cell division and RAM size and thus affects proper root development.

Since shoot meristem was also defective in seedlings grown in sirtinol medium, we also examined the expression of *CyclinB1;1:CDB-GUS* in SAM. In control, *CyclinB1;1:CDB-GUS* expression was observed in shoot meristem and developing leaf primordia (Fig. 2c). However, in sirtinol grown plants, cell division was reduced and dividing cells were randomly distributed in SAM and hypocotyl (Fig. 2c). Our results further suggest that sirtinol treatment also affects proper SAM development by affecting cell division.

### Sirtinol affects the expression of genes involved in maintenance of stem cells and meristems

**Figure 3:**
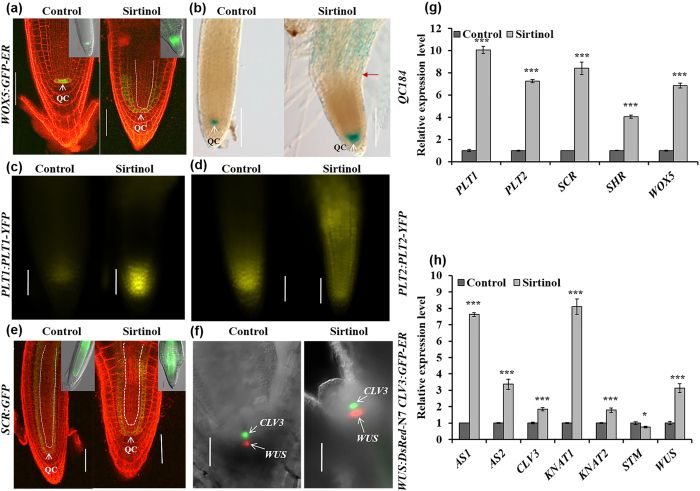
Effect of sirtinol treatment on the expression of RAM and SAM markers. (**a,b**) Sirtinol treatment causes ectopic expression of QC markers - *WOX5:GFP-ER* and QC184. Seeds of marker lines were germinated and grown on 10 μM sirtinol, GFP fluorescence (**a**) and GUS staining (**b**) was observed in seedlings at 2 dag. Scale bar: 50 μm. White dotted line in confocal image indicates a shift in the domain of *WOX5* expression. Insets in (**a**) show GFP fluorescence of same marker in DIC images. Bold red arrow marks root-shoot junction. (**c,d**) Sirtinol causes ectopic expression of *PLT1:PLT1-YFP* and *PLT2:PLT2-YFP* reporters. Seeds were germinated and grown on 10 μM sirtinol and YFP fluorescence marking the localization of PLT1/2-YFP proteins was observed in seedlings at 2 dag. Scale bar: 50 μm. (**e**) Sirtinol causes ectopic expression of *SCR:GFP*, an endodermis and QC specific marker. Seeds were germinated and grown on 10 μM sirtinol containing media, GFP fluorescence was observed in seedlings at 2 dag. Scale bar: 50 μm. White dotted lines in confocal images indicate a shift in the domain of *SCR* expression, which normally is restricted to the QC and endodermis. Insets in (**e**) show GFP fluorescence of same marker in DIC images. (**f**) Sirtinol increases *WUS* and *CLV3* expression in SAM. Seeds of *WUS:DsRed-N7 CLV3:GFP-ER* reporter line was grown on 10 μM sirtinol and fluorescence was observed in seedlings at 2 dag. Scale bar: 50 μm. (**g,h**) Sirtinol alters the expression level of genes involved in root and shoot meristem maintenance. Sirtinol treated (10 μM) seedlings (at 2 dag) were used for analysis expression level of root and shoot meristem regulatory genes (as named in labels) using real time qRT-PCR. Error bars indicate ± standard error (SE) of three independent experiments. One-way ANOVA was performed for statistical analysis. Asterisks indicate significant statistical differences, ***P < 0.001, **P < 0.01, *P < 0.05.

To investigate the effect of sirtinol on stem cells activity and meristem maintenance in root and shoot, we checked the expression of stem cell niche regulators in sirtinol grown seedlings, at 2 dag. We also performed expression analysis of the meristem specific genes using real time quantitative RT-PCR (qRT-PCR). In RAM, the expression of QC marker *WOX5:GFP-ER* was upregulated and the domain was expanded to neighboring cells, more abundantly in endodermal/cortical tissues, indicating a shift in the QC identity (Fig. 3a) caused by sirtinol treatment. We also examined the expression of another QC specific marker, QC184, in sirtinol grown seedlings. In control, QC184 was expressed in QC, whereas in sirtinol grown seedlings, its expression was prominent in QC and neighboring cells (including root stem cells) indicating that additional cells acquired quiescence (Fig. 3b).

Stem cell niche activity in the root meristem is maintained by two major parallel pathways - PLT pathway and SHR/SCR pathway. We asked if sirtinol affects SHR/SCR and PLT pathway and thus affects root meristem maintenance. We examined the expression of *PLT1* and *PLT2* using *PLT1:PLT1-YFP* and *PLT2:PLT2-YFP* reporters and observed that the expression of both PLT1-YFP and PLT2-YFP was upregulated in sirtinol treated seedlings (Fig. 3c,d and g). We also examined the expression of *SCR* using *SCR:GFP* reporter and observed that the expression of *SCR* was upregulated in sirtinol treated seedlings (Fig. 3e,g). Our expression analysis showed that the expression of *SHR* was also upregulated (Fig. 3g). Together our results suggest that sirtinol affects RAM maintenance by affecting QC identity and altering the expression of stem cell niche regulators.

Since sirtinol also affected the development of shoot meristem, we investigated its effect on the expression of shoot stem cell niche regulators by various markers and qRT-PCR analysis. We analyzed the expression pattern of *WUS* and *CLV3* using *WUS:DsRed-N7 CLV3:GFP-ER* reporter lines. The expression domain of *WUS:DsRed-N7* was expanded and transcript level was upregulated in sirtinol treated seedlings, which was also confirmed by qRT-PCR analysis (Fig. 3f,h). Interestingly, however, *CLV3:GFP-ER* also showed slightly increased expression domain and level, which was confirmed by qRT-PCR analysis (Fig. 3f,h). This indicates that sirtinol treatment creates an imbalance in WUS-CLV feedback regulatory module. SAM maintenance also requires the antagonistic activity of meristem promoting *Class I KNOX* and organ promoting *AS1/AS2* genes. We observed that the expression level of *AS1, AS2, BP/KNAT1* and *KNAT2* was upregulated and *STM* was downregulated in sirtinol treated seedlings (Fig. 3h). Based on our results, we suggest that sirtinol affects SAM activity and organ formation by altering the expression pattern of genes involved in stem cell maintenance and lateral organ formation.

### Sirtinol affects auxin and cytokinin signaling in RAM

**Figure 4:**
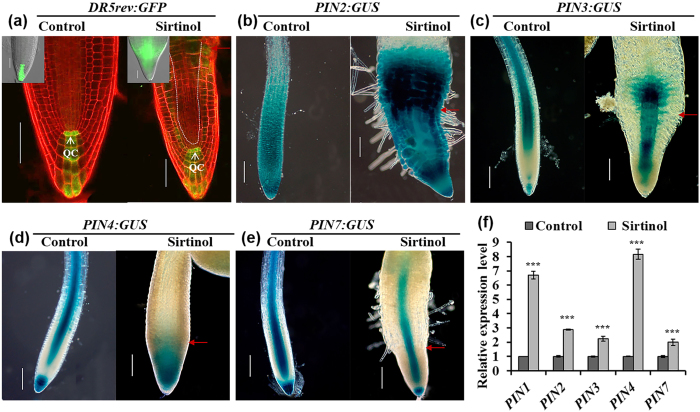
Effect of sirtinol on auxin accumulation and *PINs* expression. (**a**) Sirtinol treatment causes ectopic expression of *DR5rev:GFP* reporter, which marks auxin accumulation. To analyze the effect on auxin accumulation, seeds of *DR5rev:GFP* reporter line was grown on 10 μM sirtinol and GFP fluorescence was observed in seedlings at 2 dag. Scale bar 50 μm. White dotted lines indicate a shift in domain of *DR5rev:GFP* expression, in comparison to control. Inset in (**a**) shows GFP fluorescence analyzed by fluorescence microscope. Bold red arrow marks root-shoot junction. (**b–f**) Sirtinol alters the expression pattern of *PIN* genes in root. To analyze the effect on *PIN* gene expression pattern, *PIN2:GUS, PIN3:GUS, PIN4:GUS* and *PIN7:GUS* reporters were grown on 10 μM sirtinol and GUS staining was observed in seedlings at 2 dag. The expression levels of *PIN* genes were quantified using real time qRT-PCR in 2 days old seedlings. Bold red arrow marks root-shoot junction. Error bars indicate ± SE of three independent experiments. One-way ANOVA was performed for statistical analysis. Asterisks indicate significant statistical differences, ***P < 0.001, **P < 0.01, *P < 0.05. Scale bar 50 μm.

Since sirtinol treatment affects RAM maintenance, we were interested to know whether it alters the balance of auxin and cytokinin signaling, which are crucial component of meristem maintenance. We observed the *DR5rev:GFP* expression pattern in two days old *DR5rev:GFP* seedlings, germinated and grown in sirtinol containing media (Fig. 4a). In control, *DR5rev:GFP* was expressed in QC and columella cells (Fig. 4a). In sirtinol grown seedlings, *DR5rev:GFP* was expressed in QC, columella layers, and additionally in neighboring cells with more shootward accumulation (Fig. 4a). Auxin gradient and accumulation in the root is maintained by its transporter PIN proteins. Therefore, we checked the expression of *PINs* reporters in sirtinol grown seedlings at 2 dag. We observed that the expression of *PIN2:GUS, PIN3:GUS* and *PIN4:GUS* was induced by sirtinol treatment (Fig. 4b–d). Spatial distribution of *PIN3:GUS* and *PIN4:GUS* was also significantly altered in sirtinol treated roots (Fig. 4c,d). However, the spatial expression pattern of *PIN7:GUS* in roots of sirtinol grown seedlings remained comparable to untreated control (Fig. 4e). We further quantified the expression level by qRT-PCR analysis that confirmed the increased expression of *PIN1, PIN2, PIN3, PIN4,* and *PIN7* genes in sirtinol treated seedlings; *PIN4* showed the highest upregulation followed by *PIN1, PIN2, PIN3,* and *PIN7* (Fig. 4f). This suggests that sirtinol affects the auxin maxima and gradient formation in root meristem by altering the expression of transporter *PINs,* and thereby affects root meristem maintenance.

**Figure 5:**
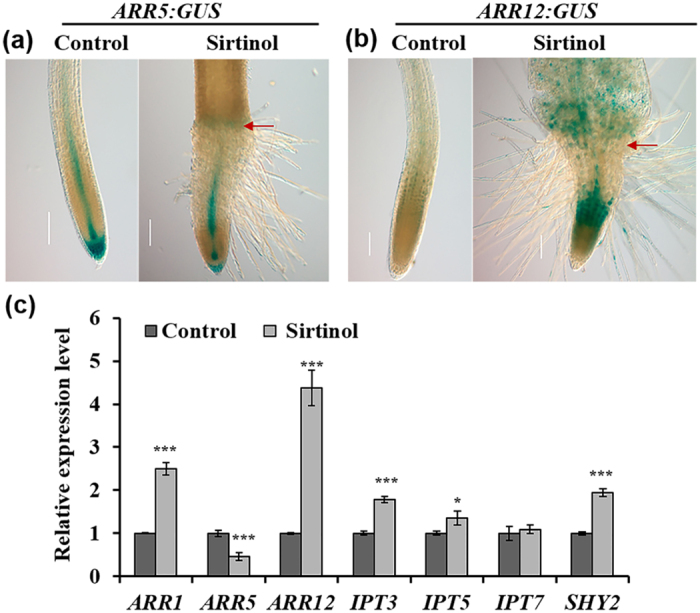
Effect of sirtinol treatment on cytokinin signaling genes. (**a**,**b**) Sirtinol alters the expression of *ARR5:GUS* and *ARR12:GUS*, cytokinin signaling markers. Seedlings were grown on 10 μM sirtinol and GUS staining was observed at 2 dag. Bold red arrow marks root-shoot junction. Scale bar: 50 μm. (**c**) Sirtinol alters the expression level of both cytokinin signaling and biosynthesis genes. Expression level of cytokinin biosynthesis and signaling genes was quantified using real time qRT-PCR in 2 days old sirtinol grown (10 μM) seedlings. Error bars indicate ± SE of three independent experiments. One-way ANOVA was performed for statistical analysis. Asterisks indicate significant statistical differences, ***P < 0.001, **P < 0.01, *P < 0.05.

To understand whether sirtinol also affects cytokinin signaling in roots, we examined the expression pattern of *ARR5* (using *ARR5:GUS* reporter), a marker of cytokinin signaling (Fig. 5a). We observed that *ARR5:GUS* expression was reduced in sirtinol grown seedlings (Fig. 5a). We also analyzed the expression pattern of positive regulator of cytokinin signaling, *ARR12*, using *ARR12:GUS* reporter and found it to be significantly upregulated in the transition zone of sirtinol grown seedlings (Fig. 5b). When quantified the expression level of cytokinin signaling genes by qRT-PCR analysis; we observed that the expression of *ARR1* and *ARR12,* and their target gene *SHY2* was upregulated, and *ARR5* was downregulated in sirtinol grown seedlings (Fig. 5c). Since we observed that the expression of positive regulators of cytokinin signaling was upregulated in sirtinol grown seedlings, we asked if cytokinin biosynthesis was also affected upon sirtinol treatment. To address this, we checked the expression of cytokinin biosynthesis genes and found that the expression of *ISOPENTENYLTRANSFERASE 3 (IPT3)* and *IPT5* was significantly upregulated upon sirtinol treatment, whereas *IPT7* expression remained unchanged (Fig. 5c). These results suggest that sirtinol affects both cytokinin signaling and biosynthesis. Our results suggest that sirtinol affects root meristem maintenance by altering not only auxin but also cytokinin signaling probably by altering the balance of auxin and cytokinin signaling.

### Sirtinol affects LR development, partially similar to auxin

**Figure 6:**
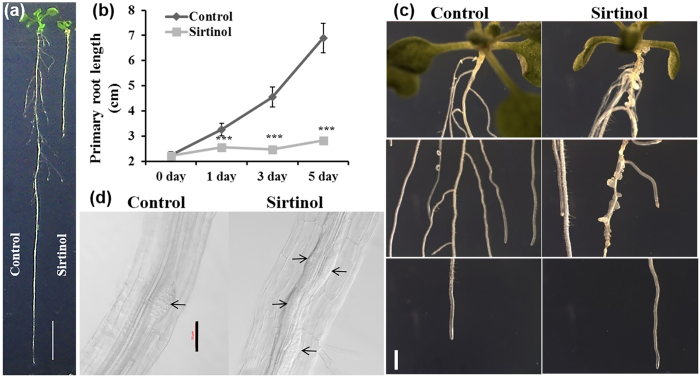
Effect of sirtinol treatment on root growth and LR formation in wild type plants. (**a**,**b**) Sirtinol inhibits primary root growth of wild type plants. To measure root growth, 5 days old wild type seedlings were transferred on sirtinol (5 μM) containing medium and grown vertically. Root length was measured at 0, 1, 3 and 5 dat. One-way ANOVA was performed for statistical analysis. Asterisks indicate significant statistical differences, ***P < 0.001, **P < 0.01, *P < 0.05. Experiment was repeated two times with reproducible results (n = 15). Scale bar: 1 cm. (**c**) Sirtinol affects LR development of wild type plants. To analyze the LR initiation and growth pattern, 5 days old wild type seedlings were transferred on sirtinol (5 μM) containing medium and LR initiation and growth was observed at 1, 3 and 5 dat. Picture depicts difference in LR development at different regions of root at 5 dat. Scale bar: 1mm. (**d**) Sirtinol causes defective LR positioning. To analyze the LR positioning defect, 5 days old seedlings were transferred to sirtinol (5 μM) and developing LRPs were marked (arrows) after 1 dat. Scale bar: 50 μm.

We have shown that sirtinol affects root meristem activity by altering both auxin and cytokinin signaling. In *Arabidopsis*, both primary and LRs constitute root architecture and auxin play an important role during LR initiation. To further identify the effect of sirtinol on later stages of root development, we treated 5 days old seedlings with 5 μM sirtinol and studied the root phenotype at different time points. We observed that sirtinol treated seedlings displayed a significant reduction in primary root length, as compared to untreated control (Fig. 6a,b). Interestingly, we found that LR development was also severely affected in sirtinol treated seedlings (Fig. 6a–d). Sirtinol treatment led to the formation of LRPs along the entire length of primary roots (Fig. 6c,d). The dividing pericycle cells led to the formation of several abnormal LRPs, which failed to emerge as proper LRs and remained like outgrowth with random divisions (Fig. 6d).

It is well established that exogenous IAA treatment leads to increased LR density and primary root growth inhibition in *Arabidopsis*. However, our results with sirtinol treatment did not show similar primary root and LR phenotype as observed in exogenous IAA (1 μM) treatment (Supplemental Fig. S2). We observed that plants treated with IAA (1 μM) formed increased number of LRs, whereas sirtinol treated plants showed abnormal LRPs which mostly did not form proper LRs (Supplemental Fig. S2a,b). These results suggest that sirtinol and exogenous IAA treatment affect LR development in a distinct manner, although they showed similar effect during the early stage of seedling growth immediately after germination.

Synthetic auxin 2,4-D, which is not a substrate for auxin efflux carriers, is known to activate cell division in all XPP cells and form abnormal LRPs along the length of the primary root^19^,^37^. To compare the effect of 2,4-D and sirtinol on LR development, we treated 5 days old Col-0 and *CyclinB1;1:GUS* seedlings with 10 μM 2,4-D and 5 μM sirtinol, and observed the root phenotype. As indicated by the expression of *CyclinB1*;*1*;*GUS* reporter, both sirtinol and 2,4-D treatment showed cell division in all XPP cells in root at 24 hrs of the treatment (Supplemental Fig. S3). 2,4-D treatment activated cell division in basal root meristem, whereas such divisions were not observed in sirtinol treated roots at 24 hrs of the treatment (Supplemental Fig. S3). Therefore, sirtinol treatment reduced the cell division in RAM (Supplemental Fig. S3). We also observed that sirtinol and 2,4-D treatment altered the expression pattern of *GATA23:GUS* reporter in both primary and LR (Supplemental Fig. S4). When treated with sirtinol and 2,4-D for 5 days, dividing XPP cells formed abnormal LRPs, which mostly did not develop into proper LRs (Supplemental Fig. S2a,b). Our results suggest that sirtinol affects LR development in a manner similar to 2,4-D. These results suggest that sirtinol may not be subjected to polar auxin transport.

### Sirtinol affects auxin accumulation and gradient formation by modulating the expression of *PIN* genes in root

**Figure 7:**
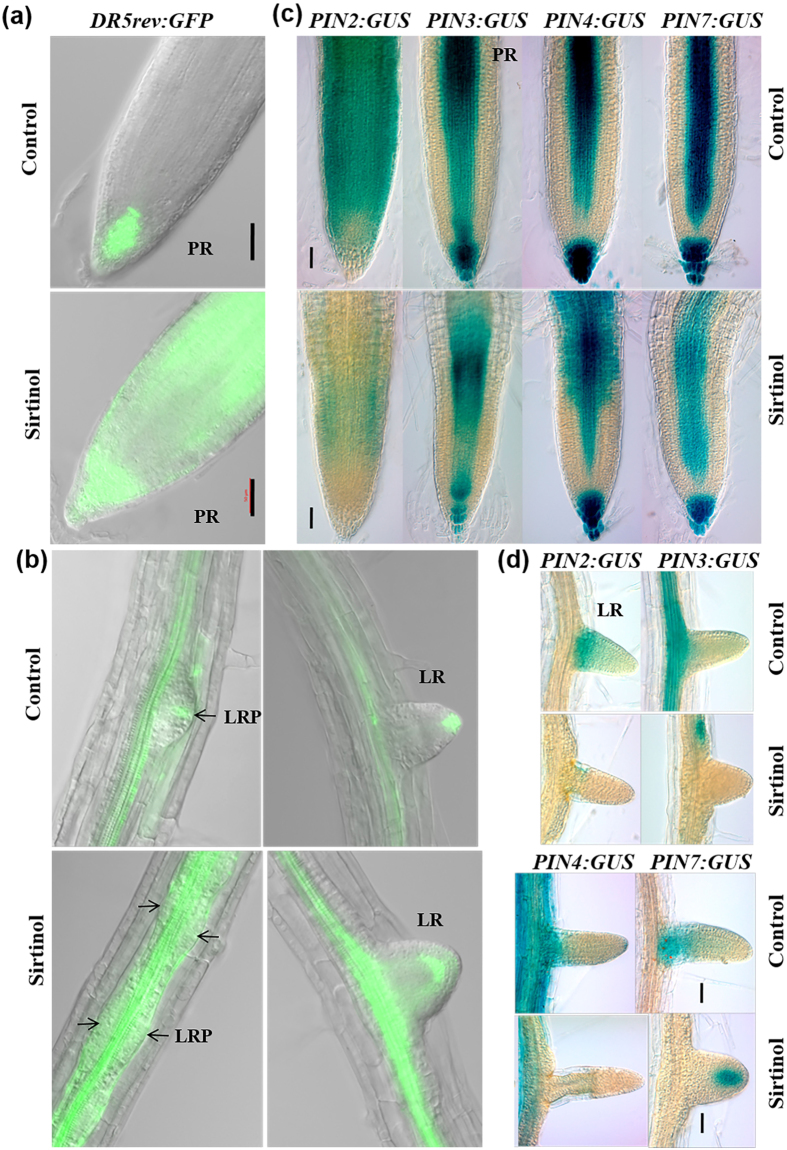
Effect of sirtinol on auxin localization and transport during later stages of root growth. (**a,b**) Sirtinol affects *DR5rev:GFP* expression in both primary root and LRs. To analyze the effect on *DR5rev:GFP* expression, 5 days old seedlings were transferred to sirtinol (5 μM) containing medium and GFP fluorescence was observed at 2 dat. Arrows show developing LRPs. Scale bar: 50 μm. (**c,d**) Sirtinol affects *PIN* genes expression in both primary root and LRs. To analyze the effect on *PIN2:GUS, PIN3:GUS, PIN4:GUS,* and *PIN7:GUS* expression, 5 days old seedlings were transferred to sirtinol containing medium (5 μM) and GUS expression was observed at 2 dat in primary root and LRs. Scale bar: 50 μm.

Since our results showed that sirtinol also affected later stages of root (primary and LR) development, we were interested to know if auxin accumulation and gradient formation were altered upon sirtinol treatment in LR developing stage of root growth. To understand this, we transferred 5 days old *DR5rev:GFP* seedlings to 10 μM sirtinol media containing plates and observed their expression in primary roots and LRs after 48 hrs of treatment (Fig. 7). As expected, primary roots showed auxin maxima near QC and columella cells in control roots (Fig. 7a). We observed that sirtinol treated seedlings rather showed a uniform distribution of auxin in primary roots (Fig. 7a). In control, the auxin accumulation was observed in tips of LRPs and emerged LRs and stele of primary root (Fig. 7b). However, in case of sirtinol treatment, auxin was uniformly distributed in LRPs, and emerged LRs also showed altered auxin accumulation (Fig. 7b). *DR5rev:GFP* showed strong expression in the stele of the primary roots of sirtinol treated seedlings (Fig. 7b). Since we observed that auxin localization was affected upon sirtinol treatment, we checked the expression of *PINs* reporters in sirtinol treated roots (Fig. 7c,d) during LR developing stage of root growth. The analysis of *PIN2:GUS, PIN3:GUS, PIN4:GUS,* and *PIN7:GUS* reporters showed their reduced expression in both primary roots and LRs (Fig. 7c,d). Together, our results suggest that sirtinol affects auxin accumulation by altering the expression of *PINs* in both primary roots and LRs, and thereby affects later stages of root development.

### Sirtinol affects the cytokinin levels and expression of genes involved in LR initiation

We have shown that sirtinol treated roots displayed defective LR development (Fig. 6c and Supplemental Fig. S3). The auxin/cytokinin ratios in the LRFCs and neighboring PCs play crucial role in LR development, besides their role in primary root growth^19^,^26^. We analyzed the expression of cytokinin biosynthesis genes and signaling genes and observed that the expression of *IPT3, IPT5* and *ARR12* was downregulated in sirtinol treated roots, suggesting that LR defects could be the result of reduced cytokinin level in roots (Supplemental Fig. S5).

Since our results showed that sirtinol affected LR formation in a manner similar to 2,4-D, we asked if these treatments affected the expression of genes involved in LR initiation. We also included treatments with other auxins - IAA and NAA to understand if sirtinol affected the expression of LR initiation genes in a manner similar or different to these auxin treatments. We treated 5 days old wild type seedlings for 2 hrs with IAA, NAA, 2,4-D (all at 10 μM), and sirtinol (5 μM) and studied the expression pattern of *ARF7, ARF19, IAA14, LBD16, LBD29,* and *GATA23* genes, which are important regulators of LR development. We observed that the *ARF19, LBD16, LBD29,* and *GATA23* were significantly upregulated in all the treatments, as compared to untreated control (Supplemental Fig. S6). *ARF7* was upregulated in all the treatments except IAA and *IAA14* was only upregulated in 2,4-D treatment (Supplemental Fig. S6). These results indicate that besides IAA, 2,4-D and NAA, sirtinol also affects the expression of LR initiation genes. Taken together our results suggest that sirtinol treatment affects LR development by affecting cell division, auxin-cytokinin balance, and expression of LR initiation genes.

## Discussion

### Sirtinol affects stem cells and meristem maintenance in *Arabidopsis*

In a chemical genetic approach, sirtinol was identified as an inhibitor of Sir2 family proteins in *Saccharomyces cerevisiae,* which also affects root and vascular tissue development in *Arabidopsis,* similar to auxin treatment^33^. Previous studies have shown that sirtinol activates auxin signaling and produces auxin related developmental phenotypes^34^,^35^. Root and shoot development is governed by the meristematic activity of RAM and SAM, which are maintained by various regulatory pathways. In this study, we have shown the effect of sirtinol treatment on stem cells and meristem maintenance. First, using a series of concentration dependent treatments, we identified that sirtinol affects plant growth in a dose dependent manner (Fig. 1). Our results have shown that sirtinol affects proper root and shoot development and alters the meristematic activity of RAM and SAM indicated by reduced cell division, as evidenced by the reduced expression of cell division marker *CyclinB1;1:CDB-GUS* (Fig. 2c and Supplemental Fig. S3). *CyclinB1;1* is a G2/M phase transition marker, which marks the actively dividing cells including RAM and SAM in *Arabidopsis*^38^,^39^. Root meristem size of sirtinol treated roots was also smaller than the untreated control (Fig. 2a,b). We suggest that sirtinol affects meristem activity of root and shoot by altering cell division pattern in both SAM and RAM.

Meristems are maintained in continuous division stage by the action of several regulatory networks, which involve hormone signaling and transcription factors, functioning in stem cell niches^7^,^40^,^41^,^42^,^43^,^44^. We observed that the expression of molecular markers or factors regulating shoot and root stem cell niches were altered in sirtinol treated seedlings. Sirtinol treatment induced ectopic expression of *WOX5:GFP-ER*, QC184, *PLT2:PLT2-YFP* and increased expression of *PLT1:PLT1-YFP* (Fig. 3). It has been shown that SCR/SHR and PLT1/PLT2 regulatory pathways play important role in the maintenance of QC and stem cell identity in *Arabidopsis* root^7^,^9^,^12^,^16^. Mutation in *WOX5* gene and loss of QC identity lead to the differentiation of distal and proximal meristem, whereas ectopic *WOX5* expression also affects quiescence and root stem cell maintenance^7^,^8^. Our results suggest that sirtinol affects QC identity, which in turn affect proper maintenance of stem cells, as evident by *PLT* expression. Since more RAM cells undergo ectopic quiescence, the cell division of stem cells and their daughters are also reduced upon sirtinol treatment, as shown with reduced *CyclinB1;1:CDB-GUS*, and this results in retarded root growth.

We observed that sirtinol treatment also affects shoot meristem maintenance. Stem cell maintenance in SAM is regulated by the independent action of *WUS*-*CLV3* module and *class I KNOX* genes^5^,^45^,^46^,^47^. WUS not only regulates the stem cell fate but it also activates the expression of its own negative regulator, *CLV3*^2^,^45^,^47^. CLV3 in a negative feedback loop restricts the *WUS* expression domain^45^,^48^. In our results, we observed that *WUS* expression domain increased upon sirtinol treatment and a slight increase in *CLV3* was also obvious (Fig. 3f,h). Our results suggest that sirtinol perturbs the WUS/CLV feed regulatory module and thus affect stem cell maintenance in SAM. In shoot, stem cell maintenance and lateral organ primordia formation occur in a fine tuned coordination. These two processes are regulated by the antagonistic interaction between class I *KNOX* genes and *AS1/AS2* genes, where STM represses *AS1* in the central zone, both AS1 and AS2 repress *KNAT1/BP* and *KNAT2* expression in flanking region of the shoot meristem^6^. Expression of *KNOX* and *AS1/AS2* genes in their action domains and repression in other’s domain controls the balance between stem cell maintenance and organ formation^6^,^49^. Our results have shown that sirtinol affects stem cell maintenance in SAM by altering the expression level and domain of SAM maintenance genes and thereby affecting organ formation as well (Fig. 3).

### Sirtinol affects RAM maintenance by modulating auxin and cytokinin signaling

We have shown that sirtinol affects RAM maintenance (Fig. 1c). It has been reported that sirtinol activates auxin signaling and increases *DR5:GUS* expression domain in *Arabidopsis*^34^. We also observed that *DR5rev:GFP* was ectopically expressed upon sirtinol treatment during early or later stages of growth, suggesting that sirtinol affects auxin localization (Fig. 4a). Our results suggest that sirtinol inhibits root stem cell maintenance by affecting auxin maxima localization, cell division and expression of root stem cell niche regulators. It has been shown previously that sirtinol is not transported through auxin polar transport, since *aux1, pin2* and *tir3* mutants behaved similar to wild type upon sirtinol treatment^34^. In this study, increased expression of *PIN1, PIN2, PIN3, PIN4,* and *PIN7* genes suggest that sirtinol alters auxin maxima formation by affecting the expression of auxin transporter PINs.

Besides auxin, cytokinin also plays important role in root development and they function antagonistically^17^,^18^. We observed that the expression of *IPT3, IPT5, ARR1, ARR12,* and *SHY2* was upregulated and *ARR5* was downregulated upon sirtinol treatment (Fig. 5). ARR1 and ARR12 are type-B response regulators, which positively regulate cytokinin signaling, whereas ARR5 is a type-A response regulator which negatively regulates cytokinin signaling^50^,^51^. Upregulation of *ARR1, ARR12* and *SHY2* suggests that sirtinol activates cytokinin signaling in roots and thus promotes differentiation of cell types. Our results suggest that sirtinol affects stem cell maintenance and root meristem activity by altering both auxin and cytokinin signaling in the root, which are also pivotal for maintaining a balance of cell division and differentiation.

### Sirtinol affects LR development similar to 2,4-D by altering auxin accumulation and transport

Several auxin signaling mutants such as *axr1, axr2, axr3,* etc. have been reported as sirtinol resistant^34^. We observed that in contrast to IAA treatment, sirtinol treated roots had several abnormal LR primordia and a few emerged LRs (Fig. 6 and Supplemental Fig. S2). It has been reported earlier that auxin transport mutants *pin2, tir3* and *aux1* were sensitive to sirtinol suggesting that sirtinol is not transported through polar auxin transport like IAA^31^. Similar to 2,4-D, sirtinol treatment led to cell division in all XPP cells (Supplemental Fig. S3). However, the cell division frequency in RAM was reduced, as compared to the control (Supplemental Fig. S3). It has been reported earlier that 2,4-D is a substrate for auxin influx carrier but not for auxin efflux carriers^37^. LR formation is largely regulated by a auxin maxima and gradient formation governed by its polar transport^25^,^52^. 2,4-D treatment leads to its accumulation in cells, as it is not secreted by efflux carriers, which affects required auxin maxima and gradient formation leading to the defective LR formation^19^,^21^. Auxin response is often monitored by *DR5rev:GFP* reporter which is expressed in founder cells and tip of developing LR primordia; auxin maxima and gradient formation in developing LRPs is largely controlled by the action of PIN proteins^21^. It has also been shown that *pin3pin7, pin4pin7, pin1pin4pin7,* and *pin1pin3pin7* combination of mutants displayed defective LR development^21^. With the help of *DR5rev:GFP* and *PINs* reporters, we observed that sirtinol also affected auxin maxima and gradient formation during LR development (Fig. 5). These results suggest that sirtinol may not be transported through polar auxin transport, which could result in its accumulation in cells, thus giving 2,4-D like phenotype during LR development.

### Sirtinol treatment affects LR formation by altering cytokinin and auxin signaling balance, and the expression of LR initiation genes

In *Arabidopsis,* LRs originate from LRFCs, which undergo several rounds of asymmetric divisions to form LRP, which eventually emerge as LR^52^. In this study, we have shown that sirtinol caused the formation of abnormal and disorganized LRPs (Fig. 6 and Supplemental Fig. S2). In *Arabidopsis*, a balance of both auxin and cytokinin regulate LR positioning^26^,^53^. In our study, we found that sirtinol treatment led to the downregulation of *IPT3* and *IPT5* in roots (Supplemental Fig. S5). Exogenously applied auxin induces cell division and leads to LR initiation along the length of the root^53^. Cytokinin has been shown to regulate the positioning of LR by inhibiting cell division in PCs neighboring LRFCs^26^. Cytokinin synthesized in cells neighboring PCs by *IPT3, IPT5,* and *IPT7* leads to the inhibition of LR formation, as was evidenced by *ipt3 ipt5* roots showing LR positioning defects^26^.

It has been shown that the perception of auxin, at the site of LR initiation, leads to the degradation of SLR/IAA14 protein resulting in the derepression of *ARF7* and *ARF19* and subsequent activation of *LBD16* and *LBD29* genes^52^,^54^,^55^,^56^. LR initiation is preceded by the founder cell specification induced by *GATA23* expression^20^,^54^. We have shown that the expression of genes of *IAA14/SLR-ARF7-ARF19* module was altered in developing LRPs upon sirtinol treatment, in a manner similar to different auxins (Supplemental Fig. S6). Thus, our results suggest that sirtinol affects root growth and LR development by modulating both auxin and cytokinin signaling in *Arabidopsis* roots.

## Methods

### Plant materials, growth conditions and chemical treatment

*Arabidopsis thaliana* accession Col-0 was used as wild type. Other seed stocks including *DR5rev:GFP*^57^, *CyclinB1;1:GUS*^39^, *CyclinB1;1:CDB-GUS*^38^, *PIN1:GUS, PIN3:GUS, PIN4:GUS, PIN7:GUS*^21^, *WOX5:GFP-ER*^58^, *QC184*^7^,^12^, *SCR:GFP*^59^, *PLT1:PLT1-YFP*^9^, *PLT2:PLT2-YFP*^9^, *WUS:DsRed-N7 CLV3:GFP-ER*^60^, *GATA23:GUS*^54^, *ARR5:GUS*^27^ and *ARR12:GUS*^61^ have been described previously. Plant growth conditions were used as described previously^55^. To study the response of sirtinol on the primary root, seeds were germinated on half MS medium supplemented with 0.01 μM, 0.1 μM, 1 μM, 2 μM, 5 μM, and 10 μM sirtinol, and growth was observed at 2 dag. For effect of sirtinol treatment on LRs, 5 days old seedlings were transferred to half MS medium supplemented with 5 μM sirtinol and phenotype was studied at different time points. We purchased sirtinol, IAA, 2,4-D and NAA from Sigma-Aldrich (https://www.sigmaaldrich.com/india.html). Primary root length was measured using ImageJ software (http://imagej.nih.gov).

### Histochemcial GUS staining and microscopy

GUS staining was performed as described previously^62^. 5-bromo-4-chloro-3-indolyl-β-d-glucuronic acid (X-gluc) was purchased from Biosynth (https://www.biosynth.com). Microscopy was done onZeiss Axio Imager 2 microscope using differential interference contrast (DIC) optics or epifluorescence (Carl Zeiss, Germany). For confocal microscopy, a Leica TCS- SP5 microscope was used (Leica microsystems, Germany). For growth studies, images were taken using a stereomicroscope (Olympus SZX16).

### RNA isolation qRT-PCR analysis

RNA isolation and real time qRT-PCR was done as described previously^63^,^64^. qRT-PCR was performed with “7900HT FAST” real time PCR system (Applied Biosystems, http://www.appliedbiosystems.com) using SYBR green based assay. Primer sequences used in the study are listed in Supplementary Table S2.

## Additional Information

**How to cite this article:** Singh, S. *et al*. Sirtinol, a Sir2 protein inhibitor, affects stem cell maintenance and root development in *Arabidopsis thaliana* by modulating auxin-cytokinin signaling components. *Sci. Rep.* **7**, 42450; doi: 10.1038/srep42450 (2017).

**Publisher's note:** Springer Nature remains neutral with regard to jurisdictional claims in published maps and institutional affiliations.

## Supplementary Material

Supplementary Figures and Tables
