# Author Correction: Sirtinol, a Sir2 protein inhibitor, affects stem cell maintenance and root development in *Arabidopsis thaliana* by modulating auxin-cytokinin signaling components

**DOI:** 10.1038/s41598-020-62385-1

**Published:** 2020-04-06

**Authors:** Sharmila Singh, Alka Singh, Sandeep Yadav, Vibhav Gautam, Archita Singh, Ananda K. Sarkar

**Affiliations:** National Institute of Plant Genome Research, Aruna Asaf Ali Marg, New Delhi, 110067 India

Correction to: *Scientific Reports* 10.1038/srep42450, published online 14 February 2017

This Article contains errors.

In Figure 1a the image for 2 μM sirtinol is a duplication of the image for 1 μM.

In addition, the legend for Figure 1,

“(**a**) Sirtinol hinders plant growth in a dose dependent manner. Wild type seedlings were grown vertically on half MS media containing 0.01 μM, 0.1 μM, 1 μM, 2 μM, 5 μM, and 10 μM sirtinol. Phenotype was observed at 2 dag. Scale bar: 1 mm. (**b**) Sirtinol leads to defective SAM and RAM. Seedlings (at 2 dag) were visualized under stereomicroscope to study the effect of sirtinol (10 μM). Scale bar: 200 μm. Black arrows indicate accumulation of starch granules (Scale bar: 10 μm).”

should read:

“(**a**) Sirtinol hinders plant growth in a dose dependent manner. Wild type seedlings were grown vertically on half MS media containing 0.01 μM, 0.1 μM, 1 μM, 2 μM, 5 μM, and 10 μM sirtinol. Phenotype was observed at 2 dag. Scale bar: 1 mm. (**b**) Sirtinol leads to defective SAM. Seedlings (at 2 dag) were visualized under stereomicroscope to study the effect of sirtinol (10 μM). (**c**,**d**) Sirtinol leads to defective RAM. Seedlings (at 2 dag) were visualized under stereomicroscope to study the effect of sirtinol (10 μM). Black arrows indicate SAM (**b**) and the accumulation of starch granules in root (**d**). Scale bar: 200 μm in (**b**-**c**); 10 μm in (**d**).”

The correct Figure 1 and its accompanying legend appear below.

**Figure 1:**
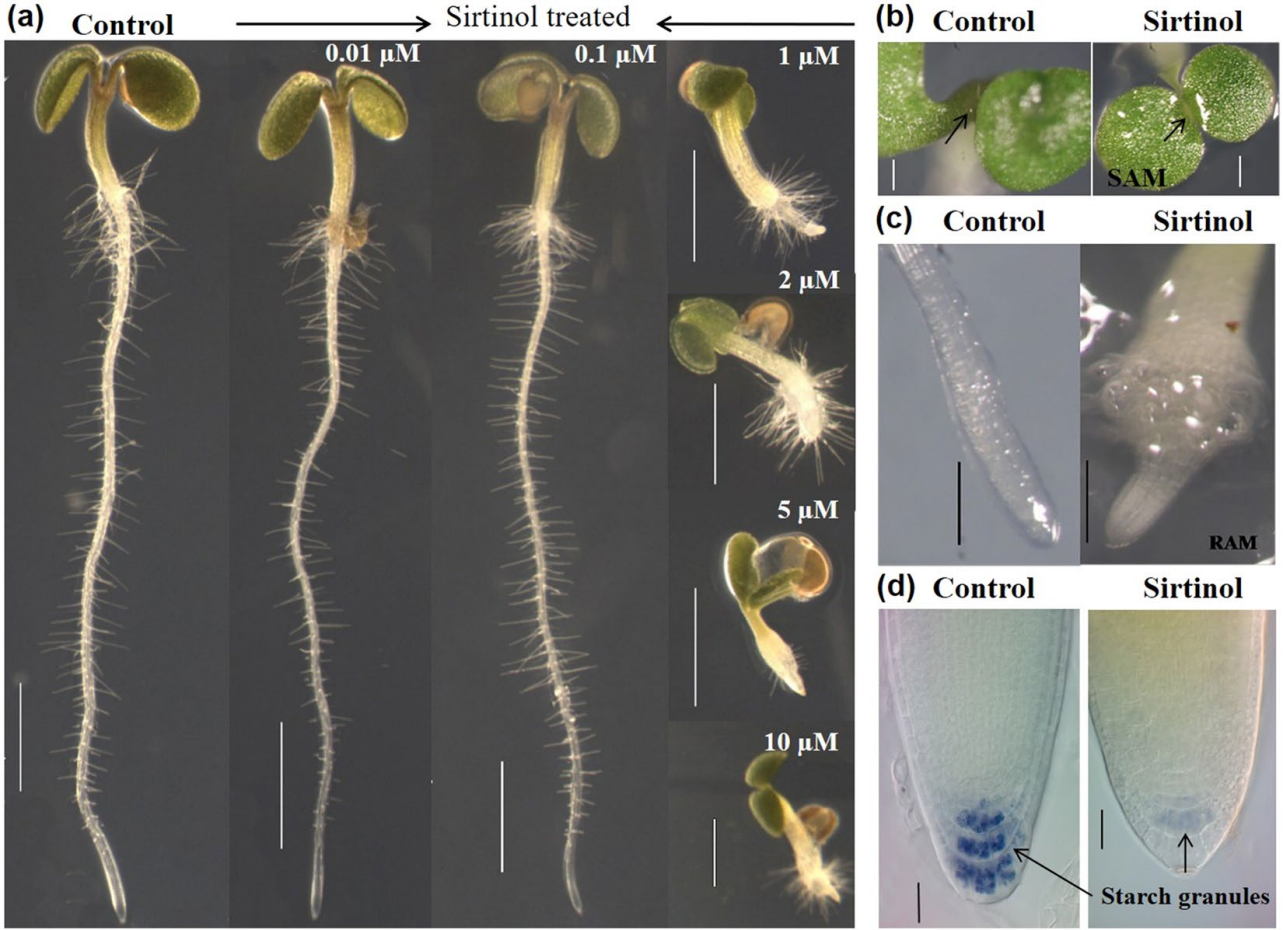
Sirtinol affects shoot and root development in a dose-dependent manner. (**a**) Sirtinol hinders plant growth in a dose dependent manner. Wild type seedlings were grown vertically on half MS media containing 0.01 μM, 0.1 μM, 1 μM, 2 μM, 5 μM, and 10 μM sirtinol. Phenotype was observed at 2 dag. Scale bar: 1 mm. (**b**) Sirtinol leads to defective SAM. Seedlings (at 2 dag) were visualized under stereomicroscope to study the effect of sirtinol (10 μM). (**c**,**d**) Sirtinol leads to defective RAM. Seedlings (at 2 dag) were visualized under stereomicroscope to study the effect of sirtinol (10 μM). Black arrows indicate SAM (**b**) and the accumulation of starch granules in root (**d**). Scale bar: 200 μm in (**b**,**c**); 10 μm in (**d**).

Furthermore, in the legend for Supplementary Figure S2,

“(c) Sirtinol affects LR development of wild type, in a manner different to IAA but similar to 2,4-D. To analyze the LR growth pattern, 5days old wild type seedlings were transferred on sirtinol (5 μM), IAA (1 μM) and 2,4-D (1 μM) containing half MS medium and LR growth was observed at 1, 3 and 5 dat. Scale bar 1 mm.”

should read:

“(b) Sirtinol affects LR development of wild type, in a manner different to IAA but similar to 2,4-D. To analyze the LR growth pattern, 5days old wild type seedlings were transferred on sirtinol (5 μM), IAA (1 μM) and 2,4-D (1 μM) containing half MS medium and LR growth was observed at 1, 3 and 5 dat. Scale bar 1 mm.”

Finally, there are typographical errors in the Primer Names for Sl. No. 11, 12, 31 and 32 in Supplementary Table S2. The correct version of Supplementary Table S2 appears below as Table 1.

**Table 1:** List of primers used in this study.

Sl. No.	Primer Name	Sequence
1	*PLT1 F*	TAGCGTCCAATCAAACGATG
2	*PLT1 R*	CGGATGGTGAAGCTTTGTC
3	*PLT2 F*	CAACGACAATATCGACAACCC
4	*PLT2 R*	CGTTGGTTTGATGAATGTCG
5	*SCR F*	CACCTACTGTATGGGTTGACG
6	*SCR R*	GAAGAGGAAGGATCAAGGAGC
7	*SHR F*	CGTGCCTTCTCCGACAAAGAC
8	*SHR R*	GTCATGCGGTTGAAGAGAGC
9	*WOX5 F*	GATTGTCAAGAGGAAGAGAAGGTGA
10	*WOX5 R*	AGCTTAATCGAAGATCTAATGGCG
11	*PIN 1 F*	TCGCTTCAGAGTTCAAGAAACC
12	*PIN 1 R*	CTCGGAGTAGGACCTTTAGAACC
13	*PIN 2 F*	CAACAAATCTCACGGCGGAG
14	*PIN 2 R*	CGTAGCTATTAGTGTAACCGTGACG
15	*PIN 3 F*	CGGGTCTTAACGTTTTCGG
16	*PIN 3 R*	TTCTCCTCCGAAATCTCCAC
17	*PIN 4 F*	TAACACTAACAGTTCTGTTCCG
18	*PIN 4 R*	CTCTTGCAGTTGCTGTTGG
19	*PIN 7 F*	CACAAGCTTCGGTGTAACTC
20	*PIN 7 R*	AAGCAACAAGAGCCCAAATG
21	*ARF7 F*	GCTCATATGCATGCTCCACA
22	*ARF7 R*	GCAATGCATCTCTGTCATATTTG
23	*ARF19 F*	CACCGATCACGAAAACGATA
24	*ARF19 R*	TGTTCTGCACGCAGTTCAC
25	*IAA14 F*	TCCTAGTTACGTGGGAATACG
26	*IAA14 R*	GGCACATTAGCATGAAGAGG
27	*GATA23 F*	TTTGATGGATCCAAGGAAGC
28	*GATA23 R*	GTCCACCTCTCCACATTGGT
29	*LBD16 F*	CGTGCGAGAGACTCATCATC
30	*LBD16 R*	TAAGAGCCAAAGCCTGAAGC
31	*LBD29 F*	TGTGCAAAGGGATGTGTGTT
32	*LBD29 R*	CGATCGCTAATGGGAAGATG
33	*KNAT1 F*	AGTCCCATTCACATCCTCAAC
34	*KNAT1 R*	ATGGTTCTTGAGTTCCCGATC
35	*KNAT2 F*	ACCGGAGACAATCAAAGACTG
36	*KNAT2 R*	TGTAGGTTTGGAGTAAGCGAGG
37	*WUS F*	GAGTAGCCATGTCTATGGATCTATGG
38	*WUS R*	CCTTCTAGACCAAACAGAGGCT
39	*CLV3 F*	CTCATGCTCACGTTCAAGGAC
40	*CLV3 R*	CTTCGTCTTTGCCTTCTCTGC
41	*AS1 F*	GTATGATGCCGTCTTGTAGTGG
42	*AS1 R*	CCTTTGTCTACACGTCTTCTCTG
43	*AS2 F*	AAGACGCAGTGAACTCTTTGG
44	*AS2 R*	GGCGAGTAAGTTGATGCAAG
45	*ARR1 F*	CGTCTGGTCTGTTGAATTGC
46	*ARR1 R*	TCCAAGCCGTCTTAGATATATCC
47	*ARR5 F*	GCTGCGAGTAGATATCATTAGCTTC
48	*ARR5 R*	GTTTGGACTGTTGAGCTGC
49	*ARR12 F*	GTTTGGACTGTTGAGCTGC
50	*ARR12 R*	ATTAGCCACACCACTGATCC
51	*SHY2 F*	AGCTGAGGCTGGGATTACC
52	*SHY2 R*	CAACAATCTGAGCCTTTCG
53	*IPT3 F*	GTGGAGGCTCTAGTGGATGAC
54	*IPT3 R*	TCTCTGACTTCCTCAACCATTCC
55	*IPT5 F*	CACCGTCCACGACACTTAC
56	*IPT5 R*	CCGGAAGTCAACGCAATC
57	*IPT7 F*	CAAGAAGTGGAAGATGTCTATGC
58	*IPT7 F*	TCCTCCGCCGTAAGATGC
59	*ACT7 F*	GGTCGTACAACCGGTATTGT
60	*ACT7 R*	GATAGCATGTGGAAGTGAGAA
61	*UBQ F*	AAGGTTCAGCGTTTGAGGAAG
62	*UBQ R*	GGATCGATCTACCGCTACAACAG

