# Supplementary material for: Sirtinol, a Sir2 protein inhibitor, affects stem cell maintenance and root development in Arabidopsis thaliana by modulating auxin-cytokinin signaling components
Source: Sci Rep. 2017 Feb 14;7:42450. doi: 10.1038/srep42450 (PMC5307962; doi:10.1038/srep42450)
Supplement: Supplementary Figures and Tables [file srep42450-s1.pdf]

Title:

Sirtinol, a Sir2 protein inhibitor, affects stem cell maintenance and root development in *Arabidopsis thaliana* by modulating auxin-cytokinin signaling components.

Authors:

Sharmila Singh[1$], Alka Singh[1$], Sandeep Yadav[1$], Vibhav Gautam[1], Archita Singh[1] and Ananda K. Sarkar[1*]

Supplemental Figures S1-S6 and Table S1, S2

Figure S1

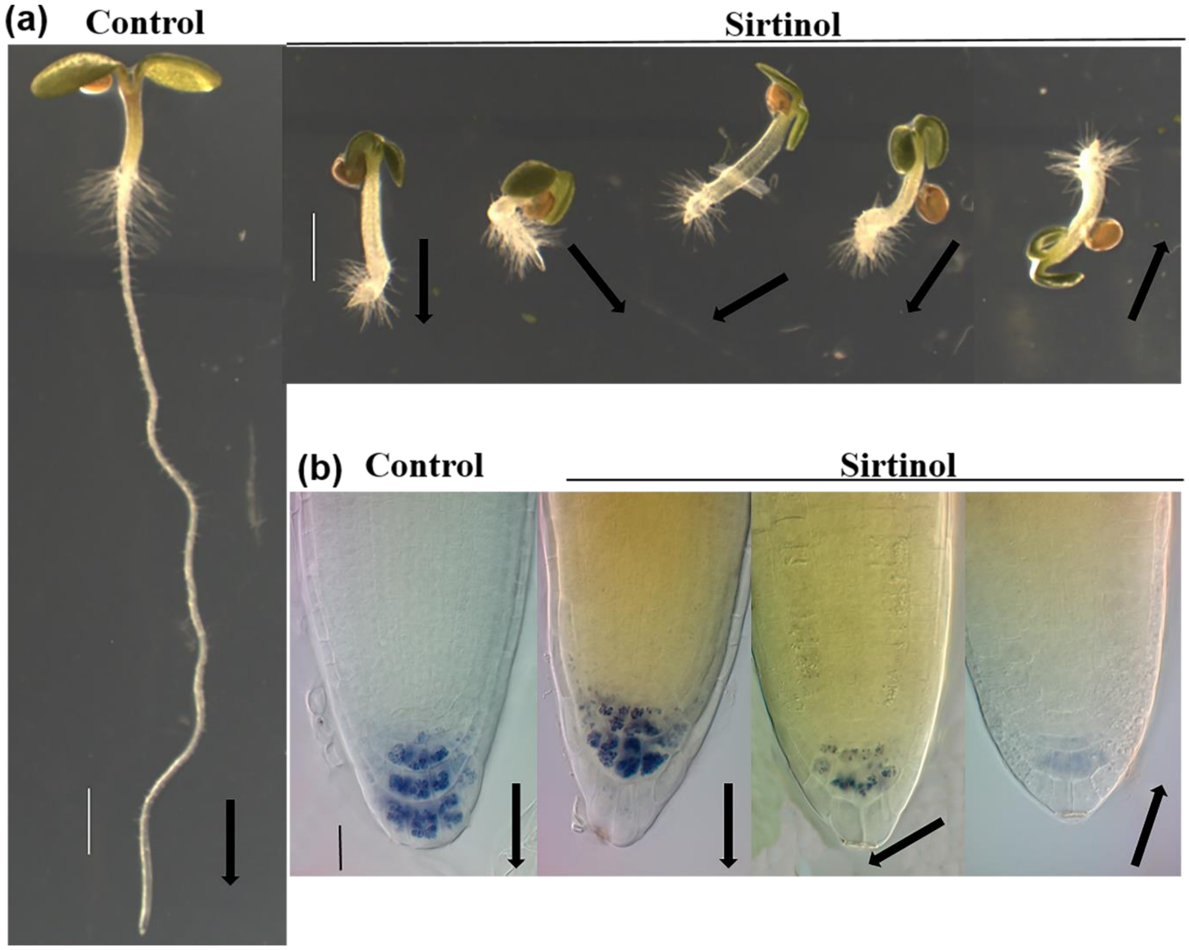

Fig. S1. Variation in root growth and gravitropic response upon sirtinol treatment.

(a) Sirtinol treatment affects the gravity response of roots. To analyze this, wild type seeds were germinated and grown on 10 µM sirtinol and their response was measured at 2 dag. Scale bar 1 mm.

(b) To check the accumulation of gravity sensing starch granules, wild type seeds were germinated and grown on 10 µM sirtinol and at 2 dag lugol staining was performed. Scale bar 10 µm. Bold black arrows depict the direction of root growth on a vertically placed control and sirtinol containing half MS media plates.

Figure S2

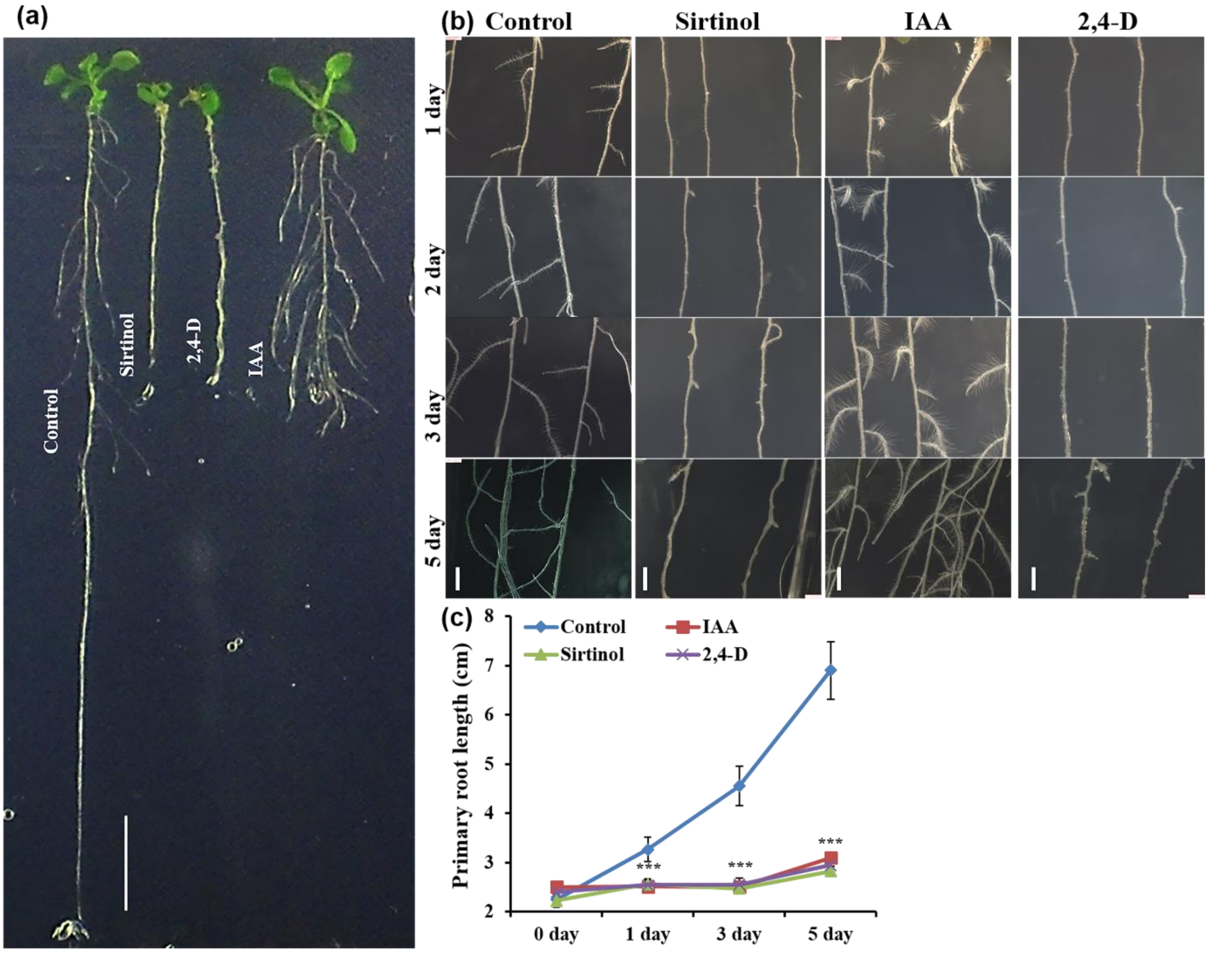

Fig. S2. Effect of sirtinol, IAA and 2,4- D on LR development.

(a,c) Sirtinol inhibit primary root growth of wild type, in 2,4-D like manner. To measure root growth, 5 days old wild type seedlings were transferred on sirtinol (5 µM), IAA (1µM) and 2,4-D (1µM) medium and root length was measured at 0, 1, 3 and 5 dat. Error bars represent ±SD (n=15). One-way ANOVA was performed for statistical analysis. Asterisks indicate significant statistical differences, ***P<0.001, **P<0.01, *P<0.05. Experiment was repeated two times with reproducible results (n=15). Scale bar 1cm.

(c) Sirtinol affects LR development of wild type, in a manner different to IAA but similar to 2,4-D. To analyze the LR growth pattern, 5days old wild type seedlings were transferred on sirtinol (5 µM), IAA (1µM) and 2,4-D (1µM) containing half MS medium and LR growth was observed at 1, 3 and 5 dat. Scale bar 1mm.

Figure S3

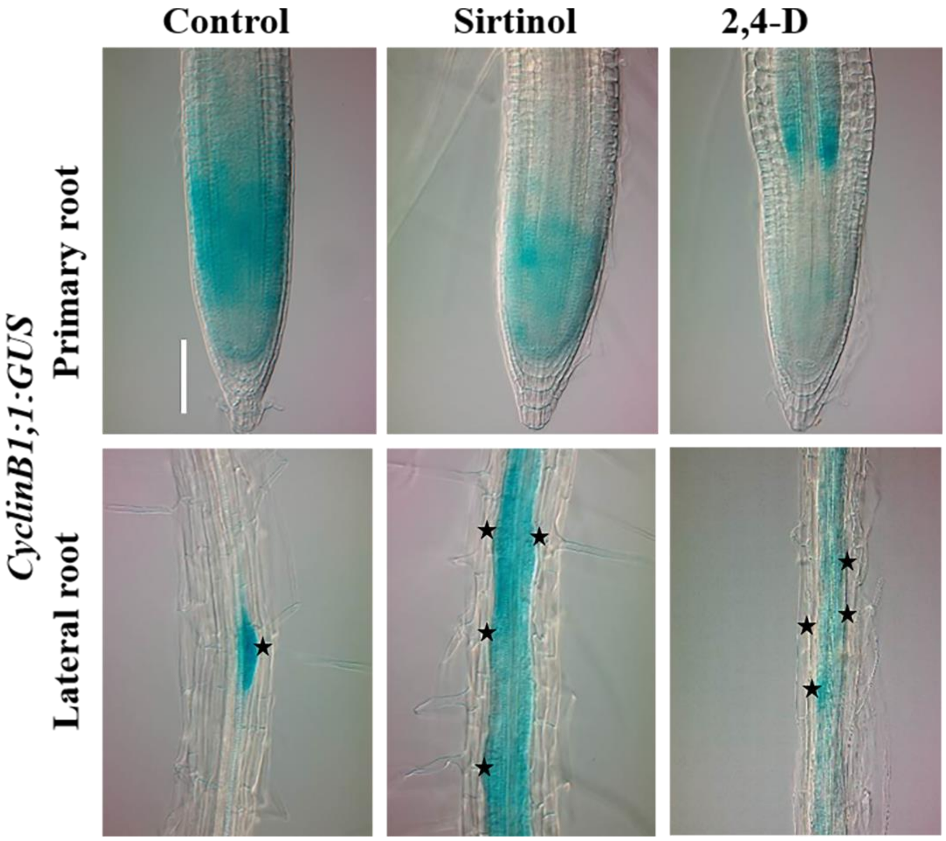

Fig. S3. Effect of sirtinol treatment on cell division during LR initiation.

Sirtinol affects LR initiation by modulating cell division in root. To analyze the cell division defect in roots, 5 days old *CyclinB1;1:GUS* seedlings were transferred to half MS, sirtinol (5 µM) and 2,4-D (10 µM) containing half MS medium plates and GUS staining was performed at 24 hrs of the treatment. Scale bar 50 µm. Black stars mark developing LRPs.

Figure S4

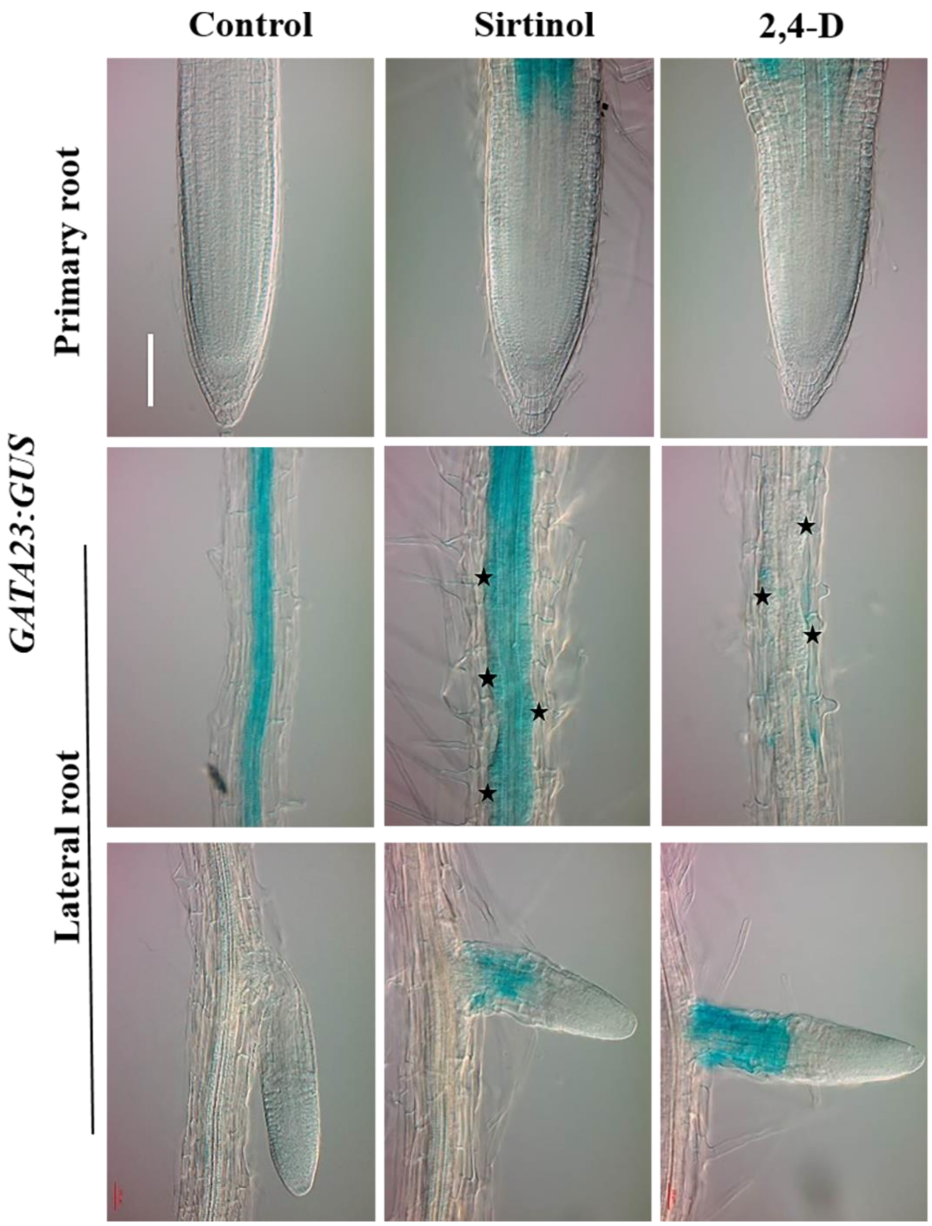

Fig. S4. Effect of sirtinol treatment on *GATA23:GUS* expression.

Sirtinol affects LR initiation by modulating cell division in root. To analyze the cell division defect in roots, 5 days old *GATA23:GUS* seedlings were transferred to half MS, sirtinol (5µM) and 2,4-D (10 µM) containing half MS medium plates and GUS staining was performed at 24 hrs of the treatment. Scale bar 50 µm. Black stars mark developing LRPs.

Figure S5

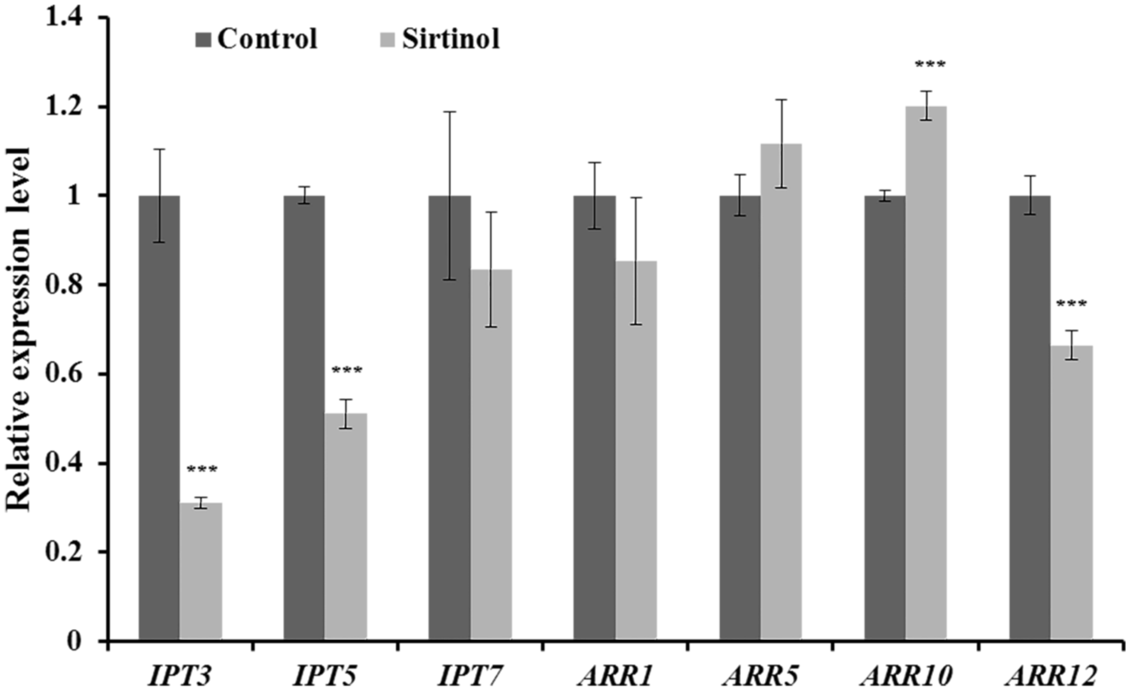

Fig. S5. Effect of sirtinol treatment on cytokinin signaling related genes.

Sirtinol affects LR spacing by inhibiting cytokinin biosynthesis and signaling genes. To analyze this, 5 days old wild type seedlings were transferred to half MS and sirtinol (5μM) containing half MS medium plates. Expression level of cytokinin biosynthesis and signaling genes was quantified using real time qRT-PCR after 2 hrs of transfer (only root tissue). Error bars indicate ±SE of three independent experiments. One-way ANOVA was performed for for statistical analysis. Asterisks indicate significant statistical differences, ***P<0.001, **P<0.01, *P<0.05.

Figure S6

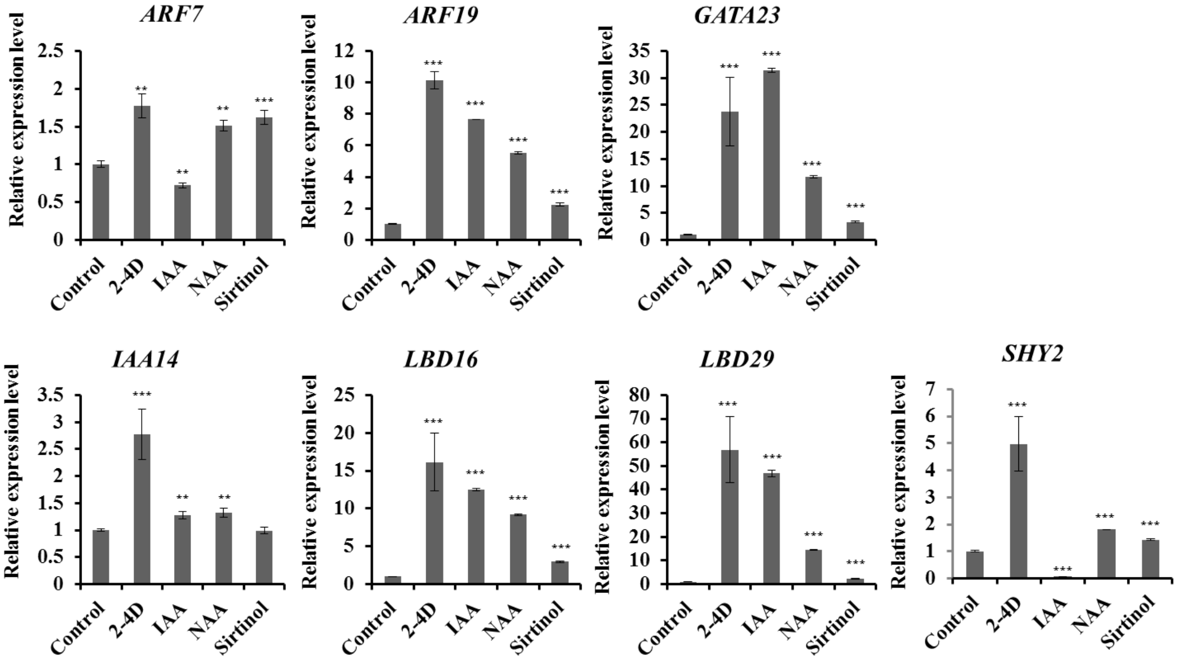

Fig. S6. Effect of sirtinol, IAA, 2,-4D and NAA treatment on LR patterning genes.

Sirtinol affects LR initiation and LR spacing. To analyze this 5 days old wild type seedlings were transferred to half MS and sirtinol (5µM), IAA (10µM), NAA (10 µM) and 2,4-D (10 µM) containing half MS medium plates. Expression level of LR pattering genes was quantified by real time qRT-PCR after 2 h of transfer (only root tissue). Error bars indicate ±SE of three independent experiments. One-way ANOVA was performed for statistical analysis. Asterisks indicate significant statistical differences, ***P<0.001, **P<0.01, *P<0.05.

Table S1. Category of sirtinol treated seedlings.

S.No.	Category	% of plants
i	Upward	28.68%
ii	Horizontal	30.28%
iii	Down	25.58%
iv	Tilted	10.85%
v	Less retarded root	5%

Table S2. List of primers used in this study.

S. No.	Primer Name	Sequence
1	*PLT1 F*	TAGCGTCCAATCAAACGATG
2	*PLT1 R*	CGGATGGTGAAGCTTTGTC
3	*PLT2 F*	CAACGACAATATCGACAACCC
4	*PLT2 R*	CGTTGGTTTGATGAATGTCG
5	*SCR F*	CACCTACTGTATGGGTTGACG
6	*SCR R*	GAAGAGGAAGGATCAAGGAGC
7	*SHR F*	CGTGCCTTCTCCGACAAAGAC
8	*SHR R*	GTCATGCGGTTGAAGAGAGC
9	*WOX5 F*	GATTGTCAAGAGGAAGAGAAGGTGA
10	*WOX5 R*	AGCTTAATCGAAGATCTAATGGCG
11	*PIN 1 P*	TCGCTTCAGAGTTCAAGAAACC
12	*PIN 1 P*	CTCGGAGTAGGACCTTTAGAACC
13	*PIN 2 F*	CAACAAATCTCACGGCGGAG
14	*PIN 2 R*	CGTAGCTATTAGTGTAACCGTGACG
15	*PIN 3 F*	CGGGTCTTAACGTTTTCGG
16	*PIN 3 R*	TTCTCCTCCGAAATCTCCAC
17	*PIN 4 F*	TAACACTAACAGTTCTGTTCCG
18	*PIN 4 R*	CTCTTGCAGTTGCTGTTGG
19	*PIN 7 F*	CACAAGCTTCGGTGTAACTC

20	*PIN 7 R*	AAGCAACAAGAGCCCAAATG
21	*ARF7 F*	GCTCATATGCATGCTCCACA
22	*ARF7 R*	GCAATGCATCTCTGTCATATTTG
23	*ARF19 F*	CACCGATCACGAAAACGATA
24	*ARF19 R*	TGTTCTGCACGCAGTTCAC
25	*IAA14 F*	TCCTAGTTACGTGGGAATACG
26	*IAA14 R*	GGCACATTAGCATGAAGAGG
27	*GATA23 F*	TTTGATGGATCCAAGGAAGC
28	*GATA23 R*	GTCCACCTCTCCACATTGGT
29	*LBD16 F*	CGTGCGAGAGACTCATCATC
30	*LBD16 R*	TAAGAGCCAAAGCCTGAAGC
31	*LBD9 F*	TGTGCAAAGGGATGTGTGTT
32	*LBD9 R*	CGATCGCTAATGGGAAGATG
33	*KNAT1 F*	AGTCCCATTCACATCCTCAAC
34	*KNAT1 R*	ATGGTTCTTGAGTTCCCGATC
35	*KNAT2 F*	ACCGGAGACAATCAAAGACTG
36	*KNAT2 R*	TGTAGGTTTGGAGTAAGCGAGG
37	*WUS F*	GAGTAGCCATGTCTATGGATCTATGG
38	*WUS R*	CCTTCTAGACCAAACAGAGGCT
39	*CLV3 F*	CTCATGCTCACGTTCAAGGAC
40	*CLV3 R*	CTTCGTCTTTGCCTTCTCTGC
41	*AS1 F*	GTATGATGCCGTCTTGTAGTGG
42	*AS1 R*	CCTTTGTCTACACGTCTTCTCTG
43	*AS2 F*	AAGACGCAGTGAACTCTTTGG
44	*AS2 R*	GGCGAGTAAGTTGATGCAAG
45	*ARR1 F*	CGTCTGGTCTGTTGAATTGC
46	*ARR1 R*	TCCAAGCCGTCTTAGATATATCC
47	*ARR5 F*	GCTGCGAGTAGATATCATTAGCTTC
48	*ARR5 R*	GTTTGGACTGTTGAGCTGC
49	*ARR12 F*	GTTTGGACTGTTGAGCTGC
50	*ARR12 R*	ATTAGCCACACCACTGATCC
51	*SHY2 F*	AGCTGAGGCTGGGATTACC
52	*SHY2 R*	CAACAATCTGAGCCTTTCG
53	*IPT3 F*	GTGGAGGCTCTAGTGGATGAC
54	*IPT3 R*	TCTCTGACTTCCTCAACCATTCC
55	*IPT5 F*	CACCGTCCACGACACTTAC
56	*IPT5 R*	CCGGAAGTCAACGCAATC
57	*IPT7 F*	CAAGAAGTGGAAGATGTCTATGC
58	*IPT7 F*	TCCTCCGCCGTAAGATGC
59	*ACT7 F*	GGTCGTACAACCGGTATTGT
60	*ACT7 R*	GATAGCATGTGGAAGTGAGAA
61	*UBQ F*	AAGGTTCAGCGTTTGAGGAAG
62	*UBQ R*	GGATCGATCTACCGCTACAACAG
